# Harnessing bioengineered microbes as a versatile platform for space nutrition

**DOI:** 10.1038/s41467-022-33974-7

**Published:** 2022-10-19

**Authors:** Briardo Llorente, Thomas C. Williams, Hugh D. Goold, Isak S. Pretorius, Ian T. Paulsen

**Affiliations:** 1 ARC Center of Excellence in Synthetic Biology, Macquarie University, Sydney, NSW 2109 Australia; 2 School of Natural Sciences, Macquarie University, Sydney, NSW 2109 Australia; 3 New South Wales Department of Primary Industries, Orange, NSW 2800 Australia

**Keywords:** Applied microbiology, Metabolic engineering, Synthetic biology

## Abstract

Human enterprises through the solar system will entail long-duration voyages and habitation creating challenges in maintaining healthy diets. We discuss consolidating multiple sensory and nutritional attributes into microorganisms to develop customizable food production systems with minimal inputs, physical footprint, and waste. We envisage that a yeast collection bioengineered for one-carbon metabolism, optimal nutrition, and diverse textures, tastes, aromas, and colors could serve as a flexible food-production platform. Beyond its potential for supporting humans in space, bioengineered microbial-based food could lead to a new paradigm for Earth’s food manufacturing that provides greater self-sufficiency and removes pressure from natural ecosystems.

## Abstract (web summary)

Long-duration human space travel creates challenges for maintaining healthy diets. Here the authors discuss using synthetic biology approaches to modify yeast into an optimal, and enjoyable, food production platform.

## Working up an appetite for food produced in space

Long-duration human space ventures, such as returning to the Moon for extended periods, visiting asteroids, and traveling to Mars, will require self-sufficiency and maximizing life support systems. Reducing dependence on initial launch cargo and resupply from Earth improves autonomy, mitigates risks, and is essential to the economic and logistic viability of long-term human endeavors in space. Biotechnology can play an integral role in achieving this goal^1,2^. Living systems are self-replicating, self-repairing, and self-organizing and can be engineered to harness available resources for the cost-effective production of desired outputs (e.g., nutrients, pharmaceuticals, biomaterials), thus having great potential to reduce cargoes and resupplies. In particular, the application of biotechnology for food-production off-Earth will be instrumental in supporting human travel and habitation through the solar system^3–5^. Here, we outline the motivation for developing microbial-based food-production systems. We examine the use of synthetic biology to consolidate multiple sensory and nutritional food attributes into engineered autotroph or waste carbon utilizing microorganisms to contribute to this effort. We also discuss the prospect of the approach in conjunction with three-dimensional (3D) printing to achieve direct consumption of fully personalized microbial food and maximize food-production with minimal waste.

## Microorganisms as space food

On short-duration missions, sufficient food can be transported for the entire mission, and regular resupplies keep the International Space Station stocked with food^3^. However, achieving food self-sufficiency will be essential for future space travelers, as the demand for food increases greatly when the duration of space endeavors lengthens^1^. By way of example, it is estimated that a mission with six crew for ~500 days of residence on Mars will require approximately five tons of food, plus another eight to ten tons for interplanetary transits and contingencies^5^. Besides the enormous logistical costs of transporting and storing large quantities of food, stored food is susceptible to deterioration, spoilage, and reductions in nutrient levels or bioavailability, which could lead to food shortages or undernutrition, compromising the crew’s health and physical and cognitive performance. Hence, other than transporting all food required or relying on Earth’s resupplies, the best approach for sustaining extended human space ventures is to produce food on-site. Ideal food systems should be capable of reliably producing appealing nutrient-rich food on-demand, fast, and with minimal inputs and physical footprint.

Different alternatives, including plants, algae, insects, cultured meat, and microbes, have been considered to produce food off-Earth^1,6–10^. Microorganisms require comparatively fewer inputs, double their biomass more rapidly, and are generally more amenable to bioengineering interventions, all of which are critical advantages that justify developing them as food-production systems. Also, besides being consumed in food and beverages like bread, yogurt, cheese, beer, and wine for millennia and, more recently, as dietary probiotics, microorganisms are increasingly used to produce food additives and are considered a sustainable food source for the future^11–13^. If microorganisms can be grown on available or waste carbon and nitrogen to produce appealing and nutritious food, then they can not only extend the duration of extraterrestrial human operations but also decrease the environmental impact of current Earth-based agriculture.

**Fig. 1:**
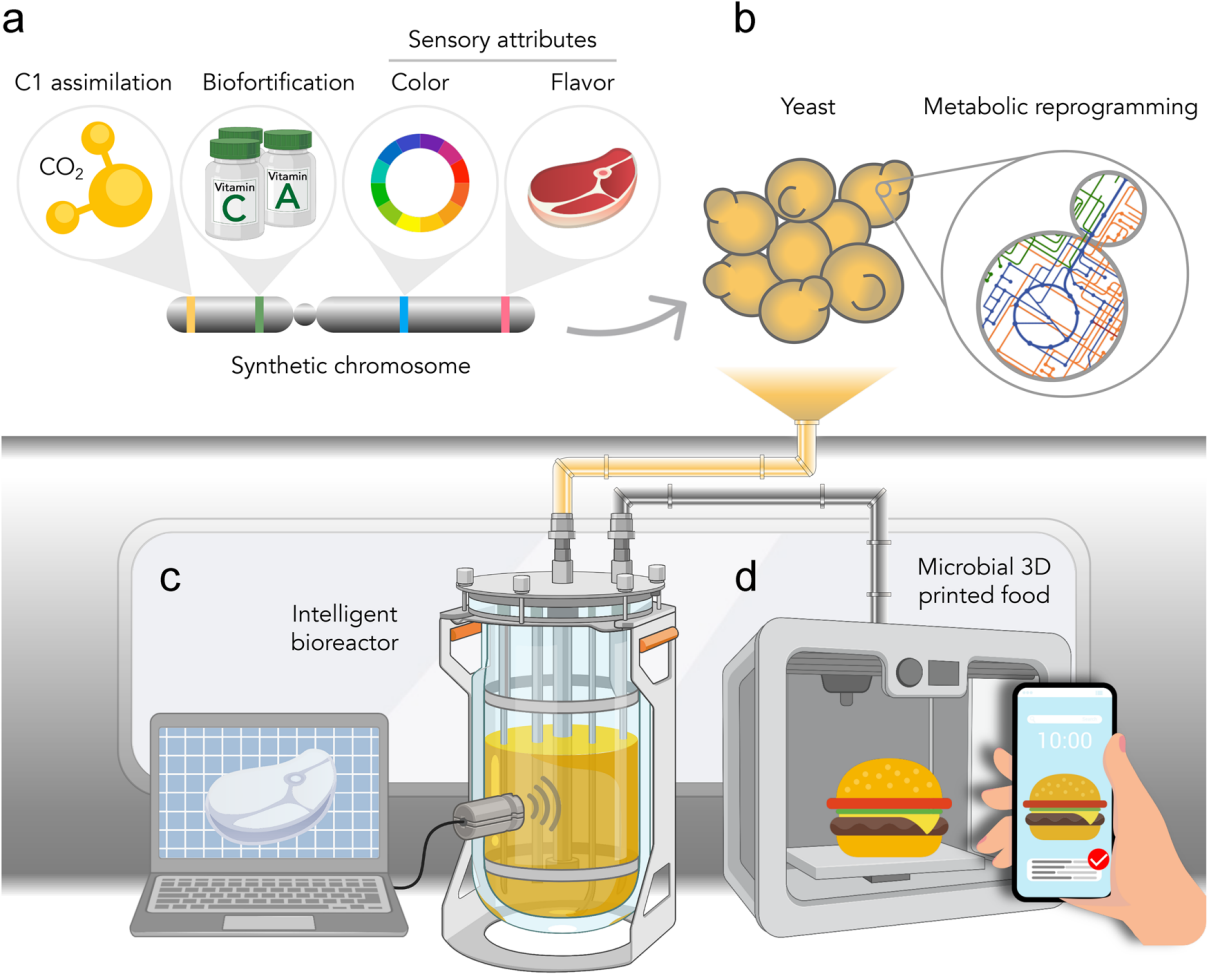
Bioengineered yeast as a complete food-production system. Multiple genetic pathways could be consolidated into synthetic chromosomes (**a**) to reprogram yeast metabolism and bestow it with new engineered traits (e.g., C1-utilization and sensory and nutritional food attributes) (**b**). Through the use of intelligent bioreactors capable of controlling the expression of specified engineered genetic pathways, yeast cellular physiology could be shaped to tune yeast biomass food properties (**c**). Microbial 3D-printed food technologies would allow manufacturing food personalized to individual preferences with minimal waste (**d**).

The yeast *Saccharomyces cerevisiae* is a food-grade microorganism with thousands of years of usage in baking, brewing, and winemaking^14^. Yeast cells are a nutritious food source and have the potential to form a more significant part of a human’s diet, as they have a macronutrient profile similar to soy flour, with ~40.4% protein, ~34.6% carbohydrates, ~1.5% lipids, and ~13 kJ per gram of dry cell weight^11,13^. The amino acid profile of yeast proteins is also suitable for human nutrition, containing all the essential amino acids that humans cannot produce and must obtain from dietary sources (i.e., histidine, isoleucine, leucine, lysine, methionine, phenylalanine, threonine, tryptophan, and valine)^15^. *S. cerevisiae* is also fast-growing (doubling time of ~90 min under optimal conditions), highly genetically malleable, and one of the most thoroughly characterized organisms^16^. Furthermore, studies have shown that microgravity conditions do not seem to significantly affect its growth or viability^17^, and work conducted by NASA has highlighted the potential for engineered *S. cerevisiae* as a useful source of essential nutrients for human health and nutrition (e.g., NASA’s BioNutrients project). Recent estimates of the nutritional value of a vitamin-prototrophic yeast strain in the context of human space travel suggest that all vitamins and macronutrients necessary for a balanced human diet could be provided for 50–100 people per day from a single 3000 L fermentation^18^. All this makes *S. cerevisiae* a promising candidate for developing into a microbial food-production system (Fig. 1).

Alternative microorganisms to *S. cerevisiae*, including naturally autotrophic ones like the cyanobacterium *Arthrospira platensis* and the alga *Chlorella vulgaris*, are already consumed as dietary supplements or food additives, and hence could be used as a food source. However, these and most other organisms are typically less well-characterized and less genetically tractable compared to yeast, meaning that engineered sensory and nutritional attributes can be challenging to implement. Another microbe with a similar depth of characterization and genetic tractability relative to yeast is the bacterium *Escherichia coli*. However, *E. coli* is arguably less suitable as a human food source due to the pathogenic nature of some *E. coli* strains and its lack of history in the human food chain.

## Recycling of essential microbial nutrients

On certain extraterrestrial destinations, such as Mars, indispensable nutrients to support microbial growth, like carbon and nitrogen, may be readily available and acquired from the environment (via in situ resource utilization)^7,19^. However, during space transits and on destinations where essential microbial nutrients are inaccessible, these resources could be partially recovered from crew-generated wastes (via closed-loop systems)^1^.

It is estimated that an average human requires about 8700 kJ of food and will expel about 740 grams of carbon dioxide (CO_2_) per day^20^. If this amount of CO_2_ could then be used to grow microorganisms in bioreactors with the nutritional content of ~12.5 kJ per gram of cells^13^, and 0.2–0.5 g of cells made per gram of CO_2_ per day^21,22^, then the total energy available from microbial food grown on recycled CO_2_ would be between 1850 and 4625 kJ per person per day. The potential utilization of different one-carbon (C1) sources using photoautotrophic or chemoautotrophic metabolism in various microorganisms was recently covered in great detail^13,23^. Assuming that waste or in situ available CO_2_ can be easily captured and released into a bioreactor, there are numerous options for growing microbial food directly on CO_2_ or by reducing CO_2_ to liquid C1 sources, such as formate or methanol. While electrochemical reduction of CO_2_ to liquid C1’s such as formate and methanol is possible, this technology is not yet mature or efficient due to the low conversion efficiencies and high energy costs^24^. Alternatively, a more mature technological possibility involves the electrochemical reduction of carbon dioxide with hydrogen to form methanol in a process already commercialized on Earth by the company Carbon Recycling International. Given that the yeast *Pichia pastoris* can grow efficiently on methanol (growth rate of 0.15/h)^25^, and there are strains of *S. cerevisiae* that can grow efficiently on ethanol made from a similar process^18^, the electrochemical reduction of CO_2_ with hydrogen poses a promising solution to the recycling of waste carbon to food in a space-travel setting. Once these problems are solved, this process could become very attractive since liquid carbon sources are easier to store and are more easily utilized in a bioreactor.

The major limitation of *S. cerevisiae* as a food-production system is its requirement for sugars as carbon and energy source. Given that the biomass yield of yeast is ~0.5 g per gram of glucose^26^, and the fact that glucose is itself a human food source, utilizing sugar-grown yeast would decrease the total calories available for human consumption. A straightforward approach to overcome this limitation would be to develop food-production systems in which yeasts are used in conjunction with autotrophic microorganisms. The simplest configuration in this paradigm would probably be for *S. cerevisiae* to grow on sugars derived from photosynthetic cyanobacteria CO_2_ fermentation or cyanobacteria biomass^27,28^. Strains of cyanobacteria have previously been engineered to secrete sugars into the growth medium^29^, which could be supplied to a second bioreactor containing sugar-utilizing yeasts, or even utilized within the same vessel through a co-culture system. Furthermore, any CO_2_ produced by yeast metabolism could theoretically be re-supplied to cyanobacteria. Alternatively, C1 assimilation could conceivably be directly engineered in *S. cerevisiae*, hence eliminating the requirement for autotrophic partners (Fig. 1).

Recent advances have allowed the functional integration of autotrophic carbon-fixing enzymes in *S. cerevisiae*^30,31^ and enabled the yeast *P. pastoris* and *E. coli* to grow directly on CO_2_, with reducing power provided by the C1 substrate formate in *E. coli*^32,33^. C1 substrates, such as formate and methanol, may also serve as carbon sources for microbial food production in space, as they can be derived readily from CO_2_ using additional oxidation and reduction reactions that are becoming inexpensive and efficient^34^. These advances make the recent engineering of formate^35^ and methanol utilization in *S. cerevisiae*^21,22,36^ relevant to microbial food production in space. While the growth rates and biomass yields of these synthetic C1-utilizing systems are still far from optimal, and co-substrates are still required, it is inevitable that they will be improved using the tools of laboratory evolution, synthetic biology, and systems biology. Although strains of *S. cerevisiae* with synthetic C1 fixation pathways may be the ultimate solution for engineered microbial food, the more immediate deployment of waste C1-utilizing microbes in space or on Earth would require the use of naturally occurring autotrophs such as hydrogenotrophic, methanotrophic, or photosynthetic microorganisms, which have been covered recently^1,23,27,28,37^.

In addition to carbon, microorganisms require a source of nitrogen. The necessary amount of nitrogen in microbial growth media is often comparable to carbon in terms of weight, making it another important consideration for long-duration space ventures and resource recycling. Human urine is one potential source of waste nitrogen that could be used for microbial fermentation, as it has high concentrations of urea (~10 g/L). Urea is a readily assimilable nitrogen source for many microorganisms, including yeast^38^. The yeast *Yarrowia lipolytica* has been grown successfully on both synthetic and natural urine^39^. Although *S. cerevisiae* can utilize urea as a sole nitrogen source, we were unable to find any reports of its growth in urine. However, given the similarity of nitrogen metabolism to *Y. lipolytica*, it is likely that *S. cerevisiae* growth can be supported in the urine-based medium. In addition to urea, human urine has other components, such as phosphates and salts, that would form part of a normal microbial growth medium, making its use particularly well-suited to support microbial food production.

It is worth noting that, although nutrient recovery from wastes to support microbial growth is feasible, bioengineered waste-reclamation systems will always be accompanied by inherent biological inefficiencies and associated energy costs. Furthermore, it is important to acknowledge that while waste recycling through synthetic microorganisms could theoretically extend a space mission, reduce the launch weight, or provide an emergency food supply, it could not do so indefinitely as there would be nonrecoverable losses of nutrients and carbon at each recycling stage.

## Developing yeast into a complete food-production system

In addition to enabling altered carbon source utilization, current synthetic biology capabilities make it possible to propose engineering multiple traits for the nutritional and sensory enhancement of *S. cerevisiae* to repurpose it for producing edible microbial biomass. We envisage that a collection of yeast strains engineered for optimal nutrition and a range of textures, tastes, aromas, and colors would help sustain a healthy diet and enable food customization to individual preferences to increase food acceptability and prevent menu fatigue, critical challenges for space food systems^3^. Such a yeast collection could be air-dried preserved (air-drying has been shown to maximize long-term yeast cell viability in space^40^) without the requirement for refrigeration and would minimize size and weight, thus being convenient for transportation and storage purposes. Selected yeast strains grown in microbial bioreactors on C1 sources or photosynthesis-derived sugars could rapidly generate edible biomass for human consumption as needed (Fig. 1).

Many strains of the proposed yeast collection could be generated by native gene knockouts and the addition of relatively small numbers of heterologous genes. However, engineering multiple genetic pathways comprising many genes and consolidating them in a minimum number of yeast strains may be challenging to achieve through classic genetic engineering approaches. One way to overcome this limitation would be to combine numerous genetic pathways of interest into synthetic neochromosomes^41,42^ designed to implement many new dietary features into yeast simultaneously (Fig. 1). We envision these dietary-dedicated neochromosomes as versatile platforms on which genetic pathways for different textures, tastes, odors, pigments, and nutrients could be added as modules depending on the characteristics of the desired strain. Designing these multi-gene modules as ‘synthetic eukaryotic operons’ (e.g., exploiting 2A peptide- or tobacco etch virus protease-based polycistronic constructs^43,44^) would allow minimized neochromosome sizes and would simplify their construction by dramatically reducing the number of promoters and terminators required.

We also foresee that strain engineering could be integrated with the development of intelligent bioreactors capable of dynamically controlling yeast biomass properties by adjusting culture conditions and cellular physiology^45,46^. Through the use of synthetic biology approaches, such as biosensors, optogenetics, and electrogenetics, these bioreactors could be programmed to orchestrate the expression of specified genes to customize yeast sensory and nutritional characteristics and produce edible microbial biomass personalized to specific requirements on demand (Fig. 1). To mitigate potential growth penalties derived from any metabolic burden caused by the activity of engineered pathways, yeast could be grown to optimal cell density only then to induce the expression of food-attributes-conveying pathways before biomass harvesting. Furthermore, once reaching the desired cell density, yeast biomass could be split into separate batches, each one induced to display particular characteristics to enable a varied menu (e.g., different meals for breakfast, lunch, and dinner) and the preparation of elaborate multi-component food (e.g., a layer cake, a burger).

## Nutritional enhancement of yeast

As discussed above, yeast is a nutritious food source for humans, having been consumed in food and beverages for thousands of years. While the extent to which a healthy human diet can consist of yeast is uncertain, dairy cattle have been fed diets with up to 40% liquid yeast cells (11% dry matter) without any negative performance or health effects^47^. Here, we propose that the nutritional value of yeast could be further enhanced to develop microbial foods for optimal human nutrition (Fig. 1).

One of the most obvious deficiencies in the nutritional profile of *S. cerevisiae* is its relatively low content of lipids (∼1.5%)^13^. A healthy human diet requires ~20–35% fats, meaning that the metabolic profile of *S. cerevisiae* would need to be modified to meet this demand if it were to comprise a significant portion of a human diet. Considerable progress has been made in increasing the lipid content of *S. cerevisiae* by combining adaptive laboratory evolution with metabolic engineering to develop yeast with high levels of fatty acids^48^. These strategies and others could be employed to contribute to the mouthfeel for microbial food and to a balanced diet, including the provision of adequate amounts of essential (i.e., linoleic acid and alpha-linolenic acid^49^) and health-promoting (e.g., omega-3 fatty acids^50,51^) fats. Alternatively, microbial species that naturally accumulate lipids, such as the oleaginous yeast *Y. lipolytica*, which is also extensively used for the commercial production of foods^52^, could be used to increase dietary fats. *Y. lipolytica* dry weight can comprise ~50% lipids^53^, and many modern synthetic biology tools are available to facilitate its metabolic manipulation^52^.

Yeast has been successfully engineered to produce many nutraceuticals^54^ and essential nutrients that humans must obtain from dietary sources, including vitamin A^55^, vitamin C^56^, and 7-dehydrocholesterol, which is directly converted into vitamin D3 in the skin upon ultra-violet (UV) light exposure^57^. Yeast production of other essential vitamins―such as vitamins B, E, and K―has not yet been fully achieved, but recent research strides in this field indicate that this may be realized in the foreseeable future^58^. Until yeast production of all essential and desired human nutrients in sufficient amounts becomes a reality, other natural or engineered microorganisms that already synthesize them^58^ could be used to supplement yeast biomass to formulate microbial food for optimal nutrition.

## Engineering food sensory attributes in yeast

Besides fulfilling human nutritional needs, if we are to develop microbial-based foods, it will be essential that edible microbial biomass also enables the production of appealing foods. In this section, we discuss the bioengineering of sensory attributes of food into microorganisms (Fig. 1).

The taste and smell sensations of food are intimately entwined and critical to our enjoyment while eating. Therefore, the engineering of microorganisms to provide a repertoire of food flavors and odors would be crucial for the development of microbial food. Fittingly, widespread applications in the food, feed, cosmetic, and pharmaceutical industries have motivated significant advances in generating taste and scent compounds in multiple yeasts^59–61^. Examples range from fruit smells, such as *S. cerevisiae* engineered to produce a ketone that imparts raspberry aroma^62^, and flavoring agents like vanillin―the key constituent of the natural vanilla flavor―produced in *S. cerevisiae* and *Schizosaccharomyces pombe*^63^, all the way through to meat flavor and aroma by the production of soy leghemoglobin in *P. pastoris*^64^.

Another key attribute of food is texture, which is highly determined by both composition and structure. Therefore, engineering *S. cerevisiae* in ways that can provide textural elements will be an important aspect for the development of yeast-based foods. Several molecules that could be used to this end have already been recombinantly produced in microbes. Examples include cellulose and starch, which are major structural and textural components of plant matter, and collagen and gelatin, which are used as textural agents in food products like yogurt and marshmallows^65–67^. In addition, as mentioned above, different approaches have also been successfully implemented to alter the lipid content of yeast^48^. Lipids not only contribute to the nutrition and taste of food, but also to texture, so altering yeast’s lipid content could also serve to modify the textural properties of yeast-based food products. Finally, there is a repertoire of biological compounds, such as pectin, lecithin, transglutaminases, and xanthan gum, that could be conceivably exploited to achieve additional textural manipulations in microbial food products.

Color is also an important sensory attribute of foods, and many microbial-derived pigments are widely used as colorants to make food products more attractive^68^. Thus, engineered pigmentation could be employed to improve the visual appeal of microbial foods. While yeast is normally a whitish beige, very different colored yeast strains can be generated by genetic manipulation. For instance, carotenoid genes can be used to impart color in the yellow to red range, while the violacein pathway and genes from bacteria and coral can be used to confer purple, green, blue, and magenta colorations^69–72^. The usage of genes for chromoprotein variants further expands the possibilities for engineering many different pigmentations^73^.

## From the bioreactor to the table

The simplest way to consume food-grade microbial biomass could probably be as a liquid or pureed homogenous food or in the form of solid food products such as noodles or snack bars. However, 3D-food printing technologies make it possible to manufacture more complex food in terms of sensory properties and to create aesthetically pleasing custom meals^74^. Hence, the development of 3D-printing methods for microbial cells could enable the realization of a plethora of different appealing food from microbial biomass. This approach could also allow maximum food output with minimal waste, as the whole edible microbial biomass harvested from bioreactors could potentially be used for printing food.

Technology for 3D printing in microgravity is being developed^75^, and it would be a powerful approach to use in conjunction with the sensory bioengineering of microorganisms to manufacture foods with a fully customized design. For instance, we imagine that microbial cells with different sensory profiles could be 3D printed to mimic the texture and appearance of vegetables or meats or into new food products of designed shapes that combine multiple types of textures and different flavors (Fig. 1).

## Future outlook

A most crucial aspect of future long-duration human space enterprises will be the development of new approaches enabling sustainable food-production off-Earth. In this context, we have considered the bioengineering of microorganisms to develop microbial-based food. We argue that the yeasts, particularly *S. cerevisiae*, pose advantages that make it attractive to repurpose them into a cornerstone microbial platform for developing a complete food-production system. Although immense hurdles remain, synthetic biology approaches offer a way to overcome yeast’s natural limitations and bestow it with tailored sensory and nutritional food attributes, as discussed above. Eventually, progress in our capacity to thoroughly engineer other microorganisms (e.g., cyanobacteria) and self-replicating synthetic cells that can be programmed to exclusively synthesize specified compounds would allow us to expand the repertoire of cell platforms for producing novel food products. This vision will also require advances in engineering infrastructure for microbial growth (e.g., microgravity conditions may require developing specific bioreactor designs and media composition) and processing (e.g., consumption of microbial biomass may require reducing nucleic acid content since, at too high concentrations, their catabolism in humans leads to accumulation of uric acid, which can cause gout-like symptoms^13^), as well as printing that interfaces with biology and has a low mass and power footprint. Noteworthy is that microbial food-production systems will not be exempt from risks that could compromise food security, such as bioreactor failure, contamination, and instability of bioengineered traits. These risks could be mitigated by implementing backup microbial stocks and infrastructure, and it is important to note that comparable challenges will likely apply to any food-production system. Experimentation onboard the International Space Station and crewed missions planned to the Moon and Mars in the next three decades^1^ provide a window of opportunity to prototype and optimize the required technologies, so they mature and become viable for supporting long-duration human presence in space.

Beyond its potential for supporting extended human endeavors in space, bioengineered microbial-based food could also open opportunities to address global food security and immediately impact Earth’s food industry. Global population growth and the associated increasing demands for food, together with the need to reduce the negative environmental impacts of modern agriculture, indicate that radical innovations will be needed to sustainably achieve food security in the coming decades. In addition to a new generation of crop plants with enhanced productivity and resilience to environmental stresses, the development of microbial foods holds enormous potential to address these challenges. The approach envisioned here could find applications in food manufacturing, food services and catering, and food retail, but also increase self-sufficiency in areas of inadequate infrastructure or in need of emergency support where local food-production capacity or availability of external food-sources is compromised (e.g., during crises, conflicts, remote places). Given the cultural importance of food and the fact that it is ingested into the body, conservatism and a high dose of skepticism will likely accompany the development of microbial food. However, the use of microbes for the industrial manufacture of feed and food-grade products is gaining prominence^37,76^, and the day may arrive soon when supermarkets and restaurants offer microbial food products, and kitchen bioreactors and microbial 3D-food printers are commonplace home appliances.
